# Comparison between landmark and ultrasound-guided techniques for internal jugular vein cannulation: A prospective observational study

**DOI:** 10.6026/973206300212946

**Published:** 2025-09-30

**Authors:** Rashmi Pal, Vijendra Singh Thakur, Rashpal Singh Gill, Harshita Dutta Prajapati

**Affiliations:** 1 Department of Anesthesiology, MGM Medical College Indore, Madhya Pradesh, India

**Keywords:** Ultrasonography, catheterization central venous, jugular veins, prospective studies, treatment outcome

## Abstract

Internal jugular vein cannulation still has complications especially with landmark (LM) technique where no imaging used. Therefore, it is of interest to compare ultrasound-guided (USG) and LM techniques in 70 patients. USG group showed better first-attempt success, shorter flash time, faster cannulation and less complication. Arterial puncture, hematoma, pneumothorax and catheter displacement were seen only in LM group proving USG safer and faster for central venous access.

## Background:

Central venous catheterization (CVC) is essential in critical care for CVP monitoring, drug and fluid infusion, blood sampling, transfusions and nutrition but still carries risks with traditional landmark-based techniques lacking imaging guidance [1]. CVC is commonly performed as either an elective or emergency procedure [2]. Internal jugular vein (IJV) is preferred for easy access and straight route to heart though subclavian and femoral veins are also used as per case and clinician preference [2, 3]. Despite extensive training, CVC placement is not without risk [4]. Complications range from minor issues, such as hematomas, to more severe outcomes, including arterial puncture, hematoma formation, pneumothorax, hemothorax, catheter displacement. Left-sided IJV cannulation can be particularly challenging, with increased risk of thoracic duct injury and longer insertion times [4, 5]. In preoperative patients IJV is reliable access especially on right side as it gives direct route to right ventricle and reduces misplacement. Two techniques used are anatomical landmark (AL) and ultrasound guided (USG). Studies show real time USG reduces complications and speeds up procedure [4]. The AL technique has long been the standard approach for IJV CVC placement, with a complication rate reported at up to 10% [2]. Factors affecting complications are patient age, body type, neck anatomy, operator skill, access site, medical condition, coagulation status and prior CVC history. Traditionally CVC is done by AL technique which is still common despite its complications [6]. Advancement in imaging especially ultrasound (USG) has given a safer option [7, 8-9]. First describe by Legler USG shows anatomy in real time and guides needle, leading to fewer attempts, faster catheterization and less complications [7, 8, 9, 10-11]. USG-guided IJV cannulation, which leverages real-time, two-dimensional ultrasound, has been shown to improve success rates and reduce complications [12]. High-frequency ultrasound probes allow clear imaging of the IJV, enabling operators to gain familiarity with cross-sectional anatomy and build confidence in needle guidance quickly and safely [13-14]. Despite advantages such as reduced complication rates and quicker procedure times, USG use in routine IJV cannulation remains limited in some clinical settings. The National Institute for Clinical Excellence (NICE) has recommended USG as the preferred method for elective IJV cannulation in adults [15]. However, adherence to this guideline is often hampered by limited access to USG machines and lack of specialized training in certain hospitals [16- 17]. Therefore, it is of interest to evaluate the benefits of USG-guided IJV cannulation in a resource-constrained ED, specifically looking at its impact on success rates, procedural duration and complication rates compared to the AL technique.

## Methods and Materials:

This prospective observational study was conducted after obtaining approval from the Institutional Ethics Committee (EC/MGM/Sept-22/50) and in accordance with the principles outlined in the declaration of Helsinki. The study was carried out for 12 months duration in the Department of Anesthesiology at MGM Medical College & M Y Hospital, Indore and Madhya Pradesh, India. A total of 70 patients were enrolled and informed written consent was obtained from all patients satisfying the inclusion criteria after explaining the study protocol in detail. The inclusion criteria included patients of age 20 to 60 years belonging to ASA Grade I, II & III and patients undergoing major surgeries under general anaesthesia requiring- (i) Central venous pressure monitoring (ii) Rapid infusion of fluids (iii) Drugs administration (iv) Blood products transfusion. The exclusion criteria included patient's refusal, SVC syndrome, Infection at the site of cannulation, Coagulopathy, Presence of carotid artery disease, contralateral diaphragmatic dysfunction, Thyromegaly, prior neck surgery and recent cannulation of internal jugular vein. Baseline data was collected using a prestructured proforma. A thorough preoperative evaluation was performed, including a general physical examination, systemic examination, assessment of the American Society of Anesthesiologists (ASA) grade, blood investigations (CBC, RBS, RFT, ECG and any specific investigations as needed) and recording of preoperative vital signs. Patients were connected to multipara monitors, which included pulse oximeter (SpO2), ECG and blood pressure monitoring. Once monitoring was established, general anesthesia induction was carried out according to standard protocols. The patients were then divided into two groups using the odd and even method, with 35 patients in each group. Group A underwent cannulation using the Landmark technique, while Group B underwent cannulation using the Ultrasound Guided technique.

## The study parameters were defined as follows:

## Success rate:

Determine by the number of attempts (≤ 3), flash time and cannulation time.

## Attempts:

Define as the introduction and removal of the introducer needle from the skin.

## Flash time:

The time intervals from skin puncture to the appearance of blood at the syringe hub.

## Cannulation time:

The time interval from the appearance of blood at the syringe hub to the confirmation of backflow in all three ports of the triple-lumen catheter. The procedure was performed under the supervision of a senior anesthesiologist. Patients requiring more than three attempts were considered failures.

## Materials used (Equipment and drugs):

The materials used in the study included a 7fr x 15 cm triple lumen central venous catheter, multipara monitor (pulse oximeter, SpO2, ECG, blood pressure), a high-frequency linear ultrasound machine (5-16 Hz), sterile drapes and towels, sponge holding forceps, suture material, Betadine solution for disinfection and emergency drugs with a crash cart.

## Procedure:

## Landmark technique:

The patient was positioned supine with a 15-20-degree Trendelenburg tilt and the neck was rotated to distend the internal jugular vein. The anatomical landmarks, including the triangle formed by the sternocleidomastoid muscle and clavicle, were identified and the carotid artery was palpated. A 20-gauge introducer needle was inserted along the medial border of the sternocleidomastoid muscle at a 30-degree angle, with aspiration of dark red blood confirming venous access. A cannula was advanced along the needle path and when free blood flow was achieved, a J-wire was inserted. After advancing the guidewire into the vein, the cannula was removed and a dilator was used to facilitate the passage of the catheter. The catheter was then threaded over the guidewire and the guidewire was removed. The catheter was secured and a sterile dressing was applied. This procedure follows the Seldinger technique, which has a high success rate and minimal complications.

## USG Guided technique:

The area was cleaned with povidone-iodine and sterile drapes were applied. The position of the internal jugular vein (IJV) was determined using a 5-16 Hz linear ultrasound probe, with the optimal view obtained from the medial oblique axis. The IJV was cannulated using a 20G introducer needle guided by ultrasound. Once proper placement was confirmed by venous blood return, a guidewire was inserted and the needle was removed. A central venous catheter was advanced over the guidewire into the IJV. The guidewire was then removed and the catheter was secured with a sterile dressing.

## Statistical analysis:

The responses were analyzed using the raw data from 70 subjects, which was entered into a computer database. Statistical analysis was performed using SPSS version 22.0. The prevalence of the outcome variable, along with 95% confidence limits, was calculated. Continuous data are presented as Mean ± Standard Deviation (Min-Max), while categorical data are presented as numbers (%). Data were categorized using a frequency distribution table. Quantitative data were expressed as mean ± SD and after checking data normality, appropriate parametric or non-parametric tests were used for analysis. The association of variables was analyzed using the Chi-square test and unpaired t-test, with a P-value <0.05 considered statistically significant.

## Results:

Total 70 patients were enrolled 35 underwent cannulation by landmark technique (Group A) and 35 by ultrasound-guided method (Group B). Males were more (47, 67.1%) than females (23, 32.9%). In Group A, 26 (74.3%) were males and in Group B, 21 (60%) were males. Mean age in LM group was 41 ± 10.32 years, while in USG group it was 38.91 ± 10.27 years. Mean BMI was 24.16 ± 2.54 in Group A and 25.36 ± 2.66 in Group B. All baseline characteristics including age, gender, BMI and ASA grade were comparable with no significant difference (P > 0.05). Most patients belonged to ASA Grade II (47, 67.1%) with no significant difference between groups (P = 0.264). Success on first attempt was significantly higher in the USG group 27 (77.1%) in Group B vs 14 (40%) in Group A. Second attempt needed in 18 (51.4%) of LM patients and 8 (22.9%) of USG group. Third attempt required only in LM group 3 patients (8.6%). All patients were eventually cannulated successfully (100%) in both groups. The number of attempts differed significantly (P = 0.004) (Table 1 - see PDF). Flash time was shorter in USG group mean 7.77 ± 1.59 sec vs 13.00 ± 2.07 sec in LM group. Cannulation time also shorter in USG group 3.02 ± 0.29 min vs 3.44 ± 0.34 min in LM group. Both differences were statistically significant (P = 0.000) (Table 2 -see PDF). Complication rate was significantly higher in LM group (Group A). Arterial puncture occurred in 28.6%, hematoma in 37.1%, pneumothorax in 14.3%, hemothorax in 8.6% and catheter displacement in 7.1%. None of these complications occurred in USG group. The difference was significant for all complications (P < 0.01) (Figure 1 -see PDF).

## Discussion:

Demographic factors like age, sex, ASA grade and BMI were similar between the groups with no significant difference (P > 0.05). Most patients were aged 40-50 years and males were more than females in 2:1 ratio. So the baseline comparability was good. But clinical outcomes showed clear benefit with USG. First attempt success was much higher with Group B (77.1%) compared to Group A (40%). Second attempts were more in LM group. Three patients in LM even needed third attempt. Statistically significant difference seen (P = 0.004). This matches findings of Turker *et al.* (2009) who showed higher attempts in landmark vs USG group (1.42 ± 0.92 vs 1.08 ± 0.33) [18]. Kunhahamed *et al.* also found more first attempt success with USG (91.4%) vs LM (48.6%) [5]. Denys *et al.* also showed USG had better first pass rate in critical care setting [19]. Flash time was shorter with USG. In our study Group B had mean flash time 7.77 sec vs 13.00 sec in Group A (P = 0.000). So access was quicker. Sazdov *et al.* similarly reported shorter access times and fewer needle passes with USG than LM. [16]. Miller *et al.* confirmed that even inexperienced operators get shorter flash time with USG [20]. Cannulation time was also better with USG 3.02 min vs 3.44 min in LM (P = 0.000). Though Kunhahamed *et al.* found no significant difference in total cannulation time (305.88 vs 293.03 sec, P = 0.425) our findings still support faster access with USG [5]. Like because once flash seen rest of steps are similar in both techniques. Complication rate was much higher in landmark group. Arterial puncture happened in 28.6% LM group, none in USG (P = 0.001). Hematoma in 28.6% LM vs none in USG (P = 0.000). Pneumothorax and hemothorax both seen in 14.3% LM group, zero in USG (P = 0.000). Catheter displacement also more in LM (8.6% vs 0%, P = 0.001) so ultrasound clearly safer. Our findings match Turker *et al.* who also reported more carotid puncture and hematoma in LM group [18]. Kayir *et al.* also showed higher complication in LM with pneumothorax 5%, hemothorax 3% also reported no difference in malposition [1]. Ahmed *et al.* highlighted the benefit of real-time USG visualization showing significant reduction in procedural errors and overall complications, supporting our findings [21]. Still our data show that USG significantly reduces procedural complications. Ultrasound helps clinician directly see vein, artery and needle all in real time. This improves accuracy and reduces risk. Literature consistently shows higher success and fewer complications with USG in CVC insertion. We defined experienced operator as someone who had done >25 successful IJV cannulations and had basic sonoanatomy knowledge. But this may not reflect true clinical experience or hand skill in real life settings.

## Conclusion:

Ultrasound-guided technique needed fewer attempts and gave faster access compared to landmark. Flash time and cannulation time both were significantly shorter with USG. Complications like arterial puncture, hematoma and pneumothorax were low with USG. USG is clearly safer and more effective than landmark for internal jugular cannulation.
